# The Appa Health App for Youth Mental Health: Development and Usability Study

**DOI:** 10.2196/49998

**Published:** 2023-10-04

**Authors:** Alison Giovanelli, Tahilin Sanchez Karver, Katrina D Roundfield, Sean Woodruff, Catherine Wierzba, J Wolny, Michelle R Kaufman

**Affiliations:** 1 Division of Adolescent and Young Adult Medicine Department of Pediatrics University of California, San Francisco San Francisco, CA United States; 2 Appa Health Oakland, CA United States; 3 Department of Health, Behavior, and Society Bloomberg School of Public Health Johns Hopkins University Baltimore, MD United States; 4 Weill Institute for Neurosciences University of California, San Francisco San Francisco, CA United States; 5 Department of Psychological and Brain Sciences Indiana University Bloomington, IN United States

**Keywords:** digital mental health, youth mentoring, cognitive behavioral therapy, teenager, adolescent, mobile phone

## Abstract

**Background:**

Demand for adolescent mental health services has surged in the aftermath of the COVID-19 pandemic, and traditional models of care entailing in-person services with licensed mental health providers are inadequate to meet demand. However, research has shown that with proper training and supervision mentors can work with youth with mental health challenges like depression and anxiety and can even support the use of evidence-based strategies like cognitive behavioral therapy (CBT). In our increasingly connected world, youth mentors can meet with young people on a web-based platform at their convenience, reducing barriers to care. Moreover, the internet has made evidence-based CBT skills for addressing depression and anxiety more accessible than ever. As such, when trained and supervised by licensed clinicians, mentors are an untapped resource to support youth with mental health challenges.

**Objective:**

The objective of this study was to develop and assess the feasibility and acceptability of Appa Health (Appa), an evidence-based mental health mentoring program for youth experiencing symptoms of depression and anxiety. This paper describes the development, pilot testing process, and preliminary quantitative and qualitative outcomes of Appa’s 12-week smartphone app program which combines web-based near-peer mentorship with short-form TikTok-style videos teaching CBT skills created by licensed mental health professionals who are also social media influencers.

**Methods:**

The development and testing processes were executed through collaboration with key stakeholders, including young people and clinical and research advisory boards. In the pilot study, young people were assessed for symptoms of depression or anxiety using standard self-report clinical measures: the Patient Health Questionnaire-8 and the Generalized Anxiety Disorder-7 scales. Teenagers endorsing symptoms of depression or anxiety (n=14) were paired with a mentor (n=10) based on preferred characteristics such as gender, race or ethnicity, and lesbian, gay, bisexual, transgender, queer (LGBTQ) status. Quantitative survey data about the teenagers’ characteristics, mental health, and feasibility and acceptability were combined with qualitative data assessing youth perspectives on the program, their mentors, and the CBT content.

**Results:**

Participants reported finding Appa helpful, with 100% (n=14) of teenagers expressing that they felt better after the 12-week program. Over 85% (n=12) said they would strongly recommend the program to a friend. The teenagers were engaged, video chatting with mentors consistently over the 12 weeks. Metrics of anxiety and depressive symptoms reduced consistently from week 1 to week 12, supporting qualitative data suggesting that mentoring combined with CBT strategies has the potential to positively impact youth mental health and warrants further study.

**Conclusions:**

Appa Health is a novel smartphone app aiming to improve the well-being of youth and reduce anxiety and depressive symptoms through web-based mentoring and engaging CBT video content. This formative research sets the stage for a large-scale randomized controlled trial recently funded by the National Institutes of Health Small Business Innovation Research program.

## Introduction

### Background

It is well established that most youth who need mental health care do not receive evidence-based services [1,2]. The COVID-19 pandemic contributed to further spikes in demand for such care [3,4] while simultaneously widening existing disparities for youth from historically underserved racial and ethnic populations, as well as lesbian, gay, bisexual, transgender, queer (LGBTQ), rural, and low-income youth [5,6]. In 2021, the US Centers for Disease Control and Prevention’s Adolescent Behavior and Experiences Survey found that one-third of high school-aged youth in the United States reported experiencing poor mental health (defined as stress, anxiety, and depression) in the past year, and 44% reported experiencing persistent periods of sadness or hopelessness [4] with higher rates for LGBTQ youth. However, youth who felt connected to others, whether in person or on the web, evinced significantly better mental health outcomes [4].

Lack of accessibility to evidence-based care continues to be a barrier for youth seeking mental health treatment, and as such, innovative solutions are needed to meet the mental health needs of teenagers [7,8]. Youth mentoring, which involves a nonparental adult or older peer building and maintaining a supportive relationship with a young person in a nonprofessional capacity [9], is one such potential solution leveraging connectedness as a protective factor. Mentors provide guidance, support, and encouragement to cultivate healthy development and outcomes. Unlike formal mental health treatment, mentoring is provided by an individual without advanced professional training (eg, a licensed therapist). There are typically fewer constraints on mentor-mentee interactions in terms of frequency, location, modality, and types of discussions between the mentor and mentee. These differences may be conducive to developing deeper personal relationships than are typical of formal mental health care and can provide a greater range of opportunities and resources for youth, particularly for those with less access to traditional care and for those who are not able to find culturally relevant care [7]. Moreover, the pool of potential youth mentors is large and relatively easily trained, which has significant implications for scalability and accessibility [10,11]. Crucially, mentors and other paraprofessionals have been found to be able to effectively support people with mental health challenges in the setting of appropriate supervision and support [11,12], with precedent for successful mentoring programs specifically targeting youth with mental health challenges [13-16]. Mentors can also serve as a bridge to connecting youth who show more serious mental health symptoms to professional care when indicated [7]. As such, youth mentoring presents a promising opportunity to reach many teenagers in need of a supportive presence.

Web-based mentoring, or e-mentoring, can further reduce barriers by meeting teenagers where they are. This modality has grown in popularity in recent years due to the proliferation of digital mobile devices, web-based accessibility, and the ubiquitous use of digital communication technology, particularly among young people [7,17,18]. As such, evidence-based e-mentoring presents an opportunity to support a greater number of young people experiencing mental health challenges.

### Appa Health: Development and Theoretical Underpinnings

Appa Health [19] is an evidence-based, theory-driven adolescent-facing mental health mobile app and mentoring program that aims to support and improve mental health for adolescents displaying clinical levels of depression or anxiety symptoms in innovative and engaging ways.

Appa Health (henceforth referred to as “Appa”), founded in 2021, was developed through an iterative process entailing close collaboration with youth and clinical advisory boards. Licensed mental health providers who are also social media content creators were contracted to develop short-form video content for its initial prototype, testing the content with the advisory boards. Youth advisors provided feedback on relatability and engagement, while clinical advisors provided feedback on accuracy and use.

This video content was then released on Appa’s platform in the form of asynchronous digital tools teaching cognitive behavioral therapy (CBT) skills. Asynchronous digital mental health tools have been found to have small but clinically meaningful impacts on youth mental health [20], but evidence suggests that adding human support to such tools in the form of guidance, encouragement, and reminders to engage can increase impacts on mental health outcomes [21-23] through both increased self-efficacy and enhanced engagement [24-26], in some cases, doubling the impacts [21-23]. Attention to engagement in particular is crucial for adolescents; while attrition rates are high for asynchronous digital interventions across age groups [26,27], consistent intervention engagement is particularly challenging in the adolescent period [28]. Through the use of mobile tools like Appa, young people have an opportunity to create meaningful relationships with relatable mentors while also receiving support to learn and generalize CBT skills in the real world, which may ultimately lead to improved mental health and quality of life.

The use of CBT tools as part of Appa’s programming is informed by extensive literature establishing CBT as a validated approach for addressing adolescent mental health symptoms [22,23], with decades of research illustrating effectiveness for treating a variety of youth concerns, including depression and anxiety, as well as general challenges with emotion regulation that contribute to many disorders [29-32]. Increased knowledge of CBT skills is a necessary foundational component [33] in increasing the use of skills, which can ultimately contribute to symptom reduction [34-36].

The theoretical basis for Appa’s combination of relatable mentor support with CBT skills is found in social cognitive theory (SCT) [37]. In SCT, a person’s beliefs about their self-efficacy, or their capabilities to plan and accomplish a given course of action, are crucial in determining behaviors like skills use. Self-efficacy can be strengthened in several ways, including through mastery experiences (eg, handling challenging situations by using a CBT skill) and vicarious experiences (eg, seeing behaviors modeled by others, particularly people who the learner admires or sees as similar to themselves in some way ). This latter aspect of SCT, known as “identification with the model” [38], is leveraged in Appa through both teaching of skills by the TikTok influencers and more explicitly through modeling examples of skills used by their near-peer mentor with shared characteristics or lived experience. In this study, we aim to examine the feasibility and acceptability of the Appa mobile e-mentoring app with an initial youth cohort.

## Methods

### Appa Health Structure and Curriculum

Appa Health is a 12-week web-based mentoring program that combines (1) short-form digital CBT content via 30-, 60-, and 90-second educational videos from mental health experts and (2) rigorously trained and closely supervised near-peer mentors with relatable lived experiences who use supportive accountability to facilitate adolescents’ learning and use of CBT skills.

#### Educational Videos

The short-form educational videos introduce and demonstrate CBT skills and concepts. Videos were filmed in collaboration with mental health content creators recruited from TikTok and Instagram. All creators were licensed psychologists, psychiatrists, or marriage and family therapists. Creators were chosen by one of two criteria: (1) either had significant existing followership on social media channels (more than 200,000 total followers) or (2) had experience distributing original mental health content through podcasts. Users were given access to 2-4 new videos each week structured around a specific CBT topic (eg, negative self-talk, cognitive distortions, and active coping strategies like progressive muscle relaxation) as well as topics like acceptance and distress tolerance.

#### Near-Peer e-Mentoring Component

Appa’s mentoring program was designed based on best practices from the existing youth mentoring research literature [7,10] with the added component of mentor compensation (US $20 per hour) due to the 10-hour weekly time commitment. Appa designed a hiring process and training program to ensure high-quality mentors are selected, onboarded, and continually monitored and supported. Training consisted of onboarding, weekly ongoing training through consultation and supervision by a licensed clinician, and quality assurance to ensure care quality and further promote mentor growth.

### Participants

Adolescent participants ages 12-18 years were recruited primarily through Appa Health promotional videos posted on social media by the mental health content creators contracted to create videos for the curriculum. Additional recruitment was facilitated via referrals from counseling departments at private high schools. Participants aged 18 years or younger (n=10) were required to have their parents begin the enrollment process in addition to parental consent to participate in this study. Participants aged 18 years or older (n=4) were able to self-enroll in the program and the study.

Exclusionary criteria included history of a psychotic disorder, history of suicidal ideation or attempt, history of self-injurious behavior, or current diagnosis of a substance use disorder. In the onboarding survey, parents were asked (1) “Has a doctor or therapist ever diagnosed your teen with a psychotic disorder like schizophrenia?” and (2) “Does your teen currently have a diagnosis of a substance use disorder?” Additionally, parents were required to affirm that their teenager did not have a history of suicidal ideation or attempt and presented no or low risk for self-injurious behavior. Participants who were aged 18 years or older were asked to affirm the same about themselves.

After participants were recruited and proceeded with enrollment, they were given the option to indicate preferences for mentor demographic characteristics, including gender, LGBTQ status, and racial or ethnic identity. Participants were then able to choose from a preselected list of available mentors that best matched their demographic preferences. After choosing a mentor, participants were given access to the Appa Health platform for a period of 12 weeks, where they could schedule weekly video sessions with their mentor, chat with their mentor via text, and watch the short-form weekly videos. Pilot study participants received the Appa Health program at no cost.

### Mentor Recruitment

In total, 10 mentors participated in the pilot study. Mentor ages ranged in age from early 20s to early 30s. All had undergraduate degrees, typically in psychology, social work, or a related field, and experience working with teenagers in contexts like residential treatment centers, summer camps, and other mentoring programs. The majority were female (n=7, 70%), and half identified as Hispanic or Latinx or people of color (n=5, 50%). Nearly one-third (n=3, 30%) of mentors identified as members of the LGBTQ community.

### Mentor Roles

Mentors were selected in part because they had relatable experiences in overcoming mental health challenges during their youth and young adult years. Mentors were trained in supportive accountability in the form of regular check-ins and problem-solving in support of participants’ learning and use of CBT strategies.

Mentors met with their youth mentees for 30 minutes weekly via Appa’s telehealth platform over the course of 12 weeks. In these meetings, they would support the teenagers in understanding and applying the video-based CBT materials released in the app over the prior week. They were also available via SMS text message within the app with the expectation that they would respond to mentee SMS text messages within 24 hours. Regular contact between parents and their teenager’s mentor is not a standard component of the Appa model, providing adolescents with an increased sense of autonomy and confidentiality in the context of rigorous safety protocols developed and overseen by licensed clinicians.

### Procedures

Adolescent participants completed surveys at baseline (week 1), midpoint (week 6), and end point (week 12) over the course of the program. Surveys took from 5 to 15 minutes to complete, depending on the timepoint. Surveys were also completed by caregivers and mentors at baseline and end point, but this study reports on adolescent survey results only. Informed assent and parental consent were obtained for all participants aged 18 years or younger (n=10), and informed consent was obtained for all participants who were 18 years of age (n=4).

Participants received US $25 in total for completing all 3 surveys and an additional US $25 for completing the short semistructured interviews at the end of the program. This study received human subject research approvals for secondary data analysis of the pilot data from the institutional review board of the Johns Hopkins Bloomberg School of Public Health.

### Measures

#### Demographics

Demographic information on the age, race, or ethnicity of the teenagers, their gender identity, and their history of receipt of mental health services was collected from both teenagers and parents.

#### Depression and Anxiety Symptoms

##### Patient Health Questionnaire-8

The Patient Health Questionnaire-8 (PHQ-8) is an 8-item self-report measure based on the PHQ-9, which was developed to assess the severity of depressive symptoms [39,40]. A cutoff of ≥10 is used to indicate clinically significant depression. PHQ-9 items are based on the 9 symptom criteria used in the Diagnostic and Statistical Manual of Mental Disorders, Fifth Edition. The PHQ-8, used in this study, excludes the ninth question asking about suicidal ideation, plan, and attempt. The PHQ-8 is commonly used in clinical research and has been found to have similar sensitivity and specificity to the PHQ-9 at the clinical cutoff of ≥10 with good reliability and validity [41,42]. Response options for each of the 8 items range from “not at all” (0) to “nearly every day” (3) for a total possible score of 24.

##### Generalized Anxiety Disorder-7

The Generalized Anxiety Disorder-7 (GAD-7) is a 7-item self-report measure based on the Diagnostic and Statistical Manual of Mental Disorders, Fifth Edition symptom criteria assessing for the presence of GAD [43,44], demonstrating good reliability and validity. Items are scored on a 4-point Likert scale ranging from “not at all” (0) to “nearly every day” (3). The GAD-7 has been validated in a large sample of adolescents and young adults ages 14-25 years; the measure demonstrated age invariance, reliability, and internal consistency (GAD-7) [44].

#### Well-Being of Youth Participants

Youth Top Problems Assessment (TPA) [45]: the TPA is a brief idiographic measure designed to identify problems that adolescents identify as especially important or impactful in their lives. Participants are asked to self-report between 1 and 3 problems in their lives in their own words, ranking the problems and then rating them in severity on a 5-point Likert scale from 0 (not a problem) to 4 (a very big problem). This measure has been validated in adolescents and has been found to be psychometrically sound [45].

#### Mentor Relationship

Mentor-Youth Alliance Scale (MYAS) [46]: the MYAS is a 9-item self-report scale assessing youth perspective on the quality of their relationship with their mentor on a 4-point Likert scale ranging from 1 (very false) to 4 (very true). The scale measures the youth-mentor bond with items like “My mentor cares about me” and “I feel comfortable with my mentor.” For the purposes of this study, 1 item was removed, and the wording of another item was altered slightly based on the needs and characteristics of the study population. The MYAS has demonstrated excellent internal consistency (0.92) and good concurrent validity [46].

#### Satisfaction With the Program

The net promoter score, or NPS, is considered the gold standard customer experience metric [47]. It is used to measure and track how a company is perceived by its customers and the loyalty of its customer base. Customers are asked the question “How likely are you to recommend this company to a friend or colleague?” measured on a Likert scale ranging from 0 (not at all likely) to 10 (extremely likely). NPS is calculated by subtracting the percentage of detractors, or participants choosing ratings of 0-6, from the percentage of promoters, or participants choosing ratings of 9 or 10. NPS scores range from −100 to +100, and while scores vary across industries, a score of 70+ is considered excellent [47].

#### In-Depth Interviews With Youth Participants

College student interns conducted short interviews with participants through the Zoom platform after school hours. Each interview was recorded through Zoom and transcribed by otter.ai (version 3.27.0; Otter.ai, Inc). Participants were all asked the same questions about topics such as (1) their general experience using Appa Health, including areas where they feel Appa Health could continue to expand and grow to better fit the needs of young people; (2) their feedback about mentoring and their relationship with their mentor; and (3) their experience navigating and using the digital content, including the “bite-sized” videos.

### Data Analysis

For quantitative survey data, responses on the PHQ-8 and GAD-7 surveys across all 3 timepoints were examined using Stata SE (version 17; StataCorp LLC). Due to the limited sample size (n=14), descriptive results in the form of frequencies, medians, and IQRs were calculated. For the quantitative aspect of the TPA, administered at the midpoint, medians and IQRs were calculated for participant rankings of the severity of each of their 3 problems.

Qualitative methods were used to examine participant responses to the free-response portion of the TPA and to the optional interviews conducted at the end of the program. For the TPA, thematic analysis was used to explore themes in participants’ free-response answers listing their top 3 problems. Thematic analysis is especially suited to assess common or shared themes presented across individuals within a data set [48]. These analyses consisted of the following steps: data familiarization, the generation of initial codes, examination of themes, revising themes, and defining and naming themes. For participant interviews, each interview was transcribed verbatim using otter.ai and then reviewed in detail. Transcribed interviews were also analyzed using a thematic analysis approach [48] applying a combination of both inductive and deductive coding. At every stage of the data analysis process, memos were used to capture any relevant data analysis insights. ATLAS.ti (version 8.4.4; ATLAS.ti Scientific Software Development GmbH) was used to manage the qualitative data.

### Ethics Approval

The secondary data analysis involving deidentified data for this research was approved by the ethical review board of the Johns Hopkins Bloomberg School of Public Health (IRB 22486). All participants whose data were used in this study provided informed consent (if 18 years of age or older) or assent with parental consent (if younger than 18 years of age) for the use of their data.

Participants were also informed about privacy and confidentiality protection with the following statement: “We will do our best to make sure that the personal information gathered for this study is kept private. However, we cannot guarantee total privacy. Your or your teen’s personal information may be given out if required by law. For example, if a condition that requires mandatory reporting (eg, a child or vulnerable adult is being abused or neglected), is discovered, the usual procedures will be followed as required by law. If information from this study is published or presented at scientific meetings, your or your teen’s name and other personal information will not be used. In addition, information from this study will only ever be presented in aggregate, such that no single individual is identifiable.” Participants were compensated with a gift card in the amount of US $25 for completion of surveys, and a subset of participants were compensated an additional US $25 if they elected to partake in a postintervention interview.

## Results

### Participant Demographics

The median age of participants (n=14) was 16 (IQR 15-18) years (Table 1). Participants were primarily white (n = 9, 64%) and female (n=12, 86%) with 14% (n=2) of teenagers identifying as male. Two participants (n = 2, 14%) identified as transgender. Parent reports indicated that half of the participants (n=7, 50%) had seen a therapist, psychologist, psychiatrist, or other mental health professional in the past.

**Table 1:** Appa Health participant sociodemographic and background characteristics at baseline.

Characteristics		Values
Age of teenagers (years), median (IQR)		16 (15-18)
**Self-reported teen gender identification, n (%)**		
	Female	12 (86)
	Male	2 (14)
	Transgender	2 (14)
**Race of teenagers,^a^ n (%)**		
	White	9 (64)
	Asian	2 (14)
	Black	2 (14)
	Mixed race: Asian and Black	1 (7)
**Previous experience of teenagers seeing a mental health professional,^b^ n (%)**		
	Yes	7 (50)
	No	5 (36)
	No response	2 (14)

^a^Based on parental and teenager report.

^b^Based on parental report.

### Feasibility and Acceptability of Appa Health

Over the course of the 12 weeks, participants engaged in video calls with their mentor for a median of 107 (IQR 50-189) minutes. The primary reasons participants reported wanting to join Appa Health were “anxiety or worries” (n=14, 100%), “sadness, low mood, or loss of interest” (n=12, 86%), and “general stress management” (n=12, 86%; Table 2). Overall, all participants (n=14, 100%) reported feeling better at the end of the 12-week program. Moreover, when asked to indicate their “main reasons for feeling better,” all participants (n=14, 100%) cited mentoring, while just over half (n=8, 57%) cited the short-form CBT videos. Consistent with this, average scores on the MYAS were high (mean 35.1, SD 0.95 of a possible 36).

The NPS, which asked participants to rate the likelihood that they would recommend Appa to a friend, was 85.75 out of a possible 100. A score of ≥70 is considered excellent [46].

**Table 2:** Feasibility and acceptability of Appa Health app.

			Values
**Primary reasons for teenagers’ decision to join Appa, n (%)**			
	Sadness, low mood, or loss of interest	12 (86)	
	Anxiety or worries	14 (100)	
	General stress management	12 (86)	
	Self-esteem	8 (57)	
	Social skills	6 (43)	
	School engagement	5 (36)	
	Loss of a loved one	2 (14)	
	Trauma	4 (29)	
	Loneliness	5 (36)	
Teenagers reporting feeling better since starting Appa, n (%)			14 (100)
**Main reasons for reporting feeling better since starting Appa, n (%)**			
	Mentoring	14 (100)	
	Videos	8 (57)	
	Letting go of the person that was effectively impacting my life	1 (7)	
	I took a lot of action in my life combined with my mentor’s advice and reassurance	1 (7)	
Net promoter score, percentage of participants indicating they would definitely recommend Appa to a friend, n (%)			12 (86)
Mentor-Youth Alliance, mean (SD)			35.1 (0.95)
Time engaged in video calls with mentor (minutes), median (IQR)			107 (150-189)

### Depression and Anxiety Symptoms

#### Patient Health Questionnaire-8

**Figure 1:**
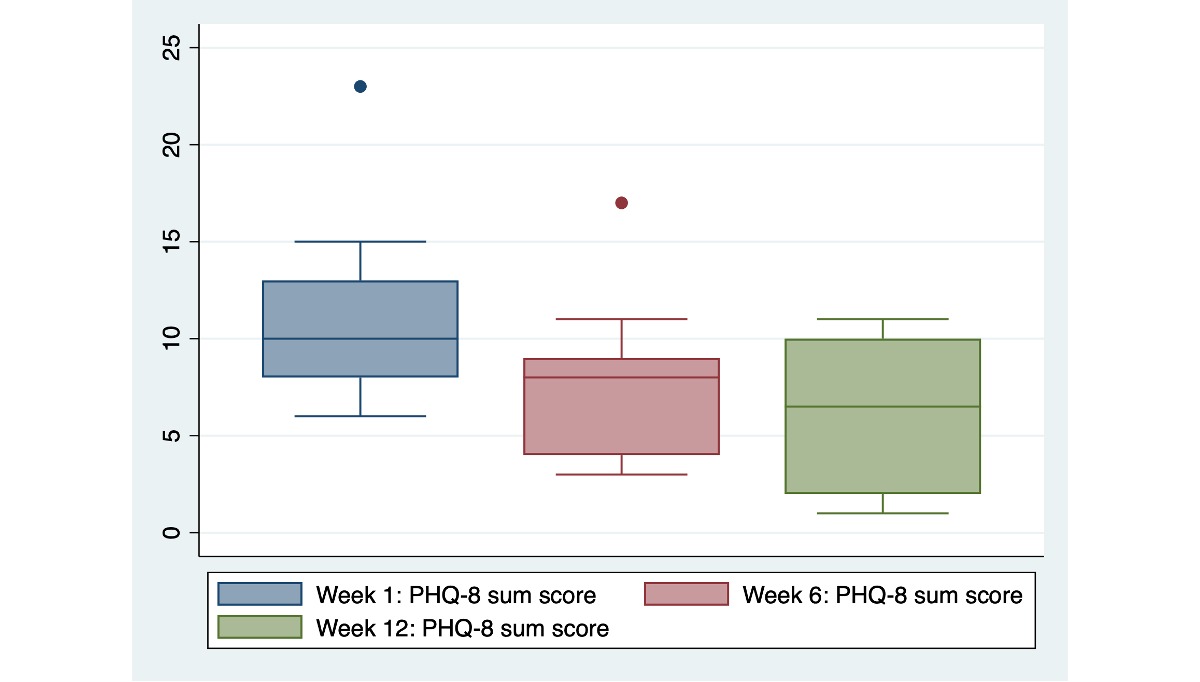
Appa Health participant PHQ-8 scores by week (N=14). PHQ-8: Patient Health Questionnaire-8.

Median scores on the PHQ declined from 10 (IQR 8-13) at week 1 to 6.5 (IQR 2-10) at week 12 (Table 3 and Figure 1). Categories of depressive symptom severity were also examined. At week 1, 50% (n=7) of participants reported moderate, moderately severe, or severe depressive symptoms. By week 12, 29% (n=4) of participants reported moderate depressive symptoms with no participants reporting moderately severe or severe symptoms. Moreover, 29% (n=4) reported no significant depressive symptoms at week 12.

**Table 3:** Participants’ mental health scores and classification by weeks (N=14).

					Values
**PHQ-8^a^ score, depressive symptoms, median (IQR)**					
	Week 1			10 (8-13)	
	Week 6			8 (4-9)	
	Week 12			6.5 (2-10)	
**GAD-7^b^ score, anxiety symptoms, median (IQR)**					
	Week 1			8.5 (7-11)	
	Week 6			6.5 (5-8)	
	Week 12			6.5 (4-7)	
**PHQ-8 depression classification, n (%)**					
	**Week 1**				
		Mild depressive symptoms	7 (50)		
		Moderate depressive symptoms	5 (36)		
		Moderately severe depressive symptoms	1 (7)		
		Severe depressive symptoms	1 (7)		
	**Week 6**				
		No significant depressive symptoms	4 (29)		
		Mild depressive symptoms	7 (50)		
		Moderate depressive symptoms	2 (14)		
		Moderately severe depressive symptoms	1 (7)		
	**Week 12**				
		No significant depressive symptoms	4 (29)		
		Mild depressive symptoms	6 (43.)		
		Moderate depressive symptoms	4 (29)		
**GAD-7 anxiety classification, n (%)**					
	**Week 1**				
		Mild anxiety symptoms	9 (64)		
		Moderate anxiety symptoms	4 (29)		
		Severe anxiety symptoms	1 (7)		
	**Week 6**				
		Minimal anxiety symptoms	2 (14)		
		Mild anxiety symptoms	9 (64)		
		Moderate anxiety symptoms	1 (7)		
		Severe anxiety symptoms	2 (14)		
	**Week 12**				
		Minimal anxiety symptoms	5 (36)		
		Mild anxiety symptoms	9 (64)		

^a^PHQ-8: Patient Health Questionnaire-8.

^b^GAD-7: Generalized Anxiety Disorder-7.

#### Generalized Anxiety Disorder-7

**Figure 2:**
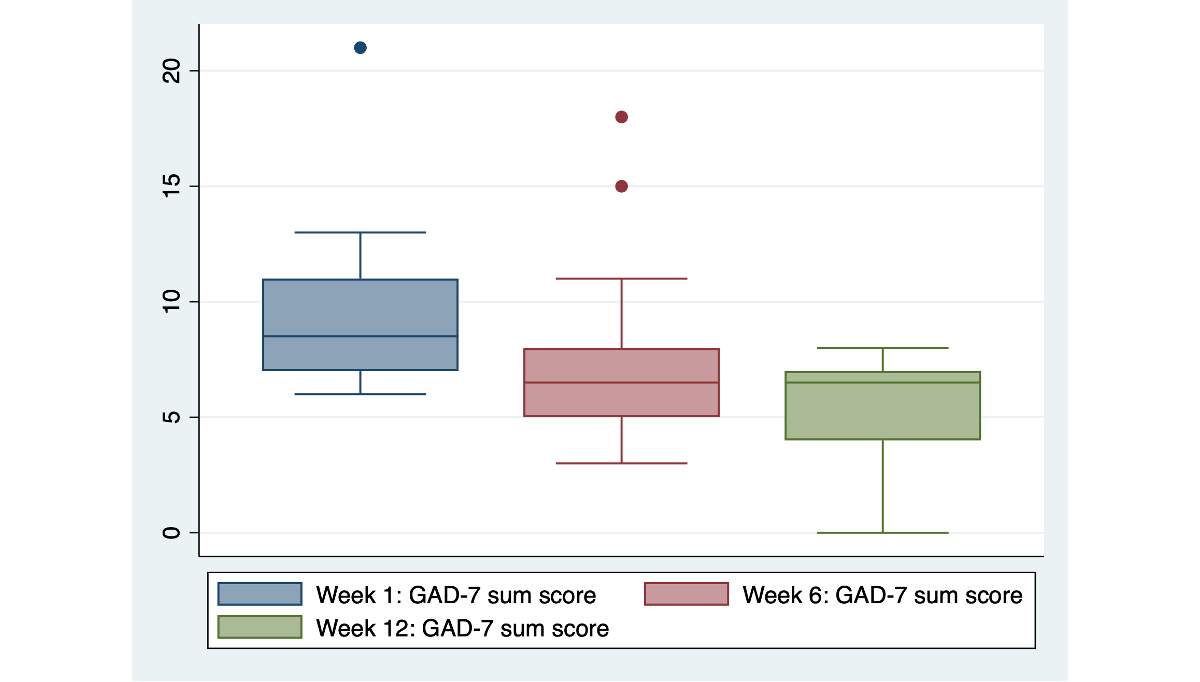
Appa Health participant GAD-7 Anxiety scores by week (N=14). GAD-7: Generalized Anxiety Disorder-7.

Median GAD-7 scores reduced from 8.5 (IQR 7-11) at week 1 to 6.5 (IQR 4-7) at week 12 (Table 3 and Figure 2). At week 1, about one-third (n=5, 36%) of participants scored in the moderate or severe anxiety category on the GAD-7. By week 6, that number had dropped to 21% (n=3), and by week 12, no participants endorsed moderate or severe anxiety symptoms.

### Youth Top Problems

**Figure 3:**
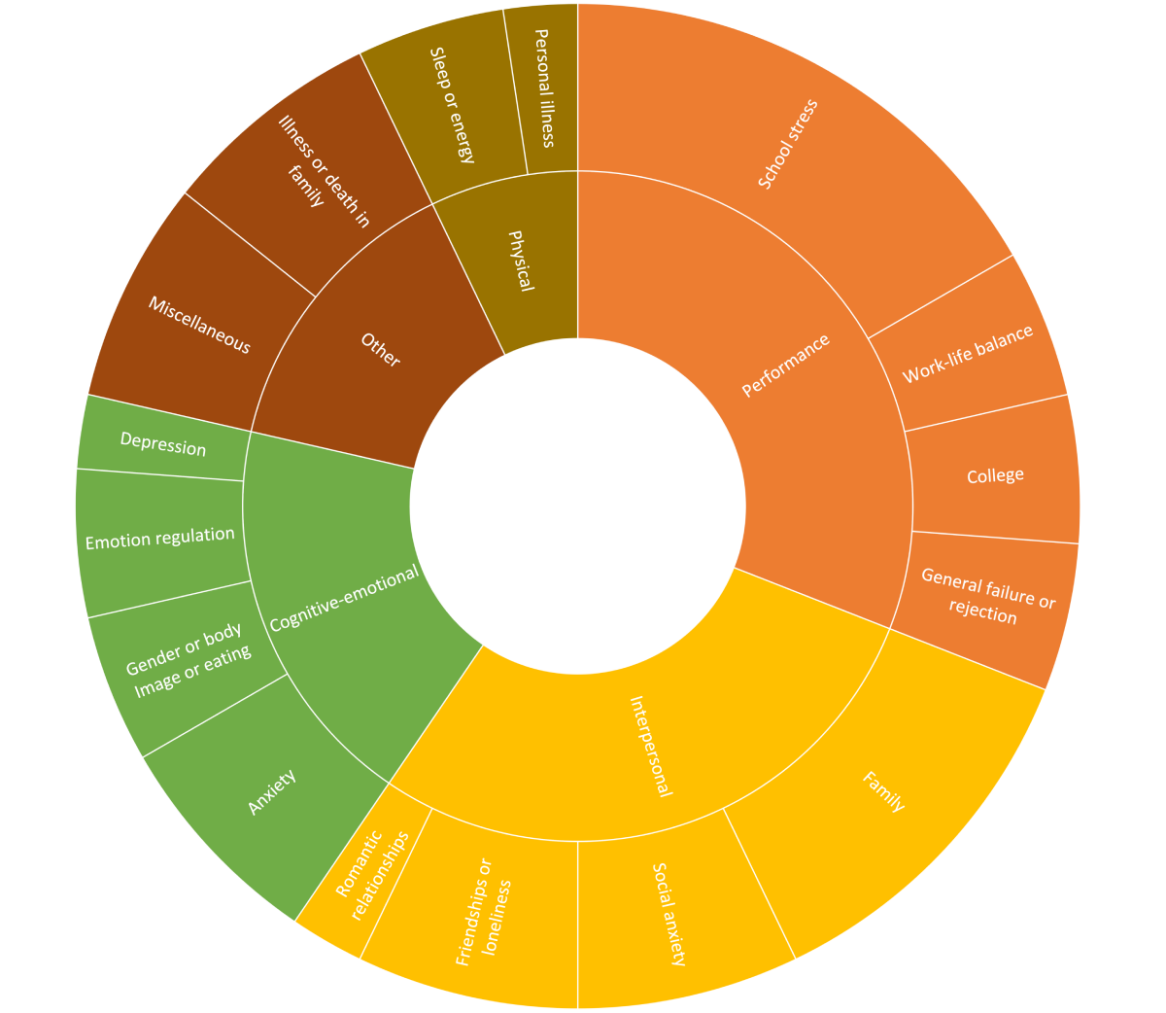
Appa Health participant report of Youth Top Problems at midpoint (participant: n=14 and problems: n=42).

Each of the 3 problem areas received similar severity ratings across participants (TPA 1: median 3, IQR 3-4; TPA 2: median 3, IQR 2-3; and TPA 3: median 3, IQR 2-4; Table 4 and Figure 3) [45]. These problems fell into 5 broad themes: *performance, interpersonal*, *cognitive or emotional*, *physical*, and *other* (Figure 3), with most problems reported falling in the *performance* (n=13, 31%) and *interpersonal* (n=12, 29%) categories. Accordingly, the majority of participant-reported problems were framed in terms of functional outcomes, particularly, as related to performance (eg, school and college applications) and interpersonal relationships (eg, friendships, family, and romance). Thus, participants were largely concerned with issues impacting daily living (eg, “stressed about schoolwork” and “loneliness”). The third most endorsed theme of cognitive or emotional (n=8, 19%) problems demonstrated that many participants also conceptualized their issues in terms of the cognitive and emotional processes driving their issues (eg, “trouble regulating emotion” and “overwhelming anxiety, the constant feeling and worry that I’m not doing enough, that I’m running out of time, and that I’m watching my life rush by”).

**Table 4:** Top Problems Assessment ratings (N=14).

	Values, median (IQR)
Problem 1	3 (3-4)
Problem 2	3 (2-3)
Problem 3	3 (2-4)

### Semistructured Interviews With Teenaged Participants

Teenagers were given the option to participate in short semistructured interviews after completion of the program. Interviews with 5 teenagers ranged in length from 10.52 to 25.37 minutes with a median length of 14.38 (IQR 11.52-17.18) minutes.

### Overall Perspective on Appa Health

Participants were asked to think of the first 3 words that come to mind when they think about Appa Health. All participants expressed positive feelings about Appa Health, with 4 of the 5 participants choosing “support” as one of their 3 words. Other words shared by participants included “comfort,” “friendship,” “healthy,” “proactive,” “text,” and “caring.” One participant further elaborated on this response and stated:

[Three words to describe Appa are] supportive, engaging, and... just like, basically like providing the tools I need to, like, build a better mental health space around me. And... equipping me with the proper strategies and concepts

Cisgender male participant, 18 years old

### Addressing Barriers to Access Teenage Mental Health Services

For 3 of the 5 participants, Appa Health presented a new opportunity to access teenage mental health services using a different modality from traditional, in-person, services. In total, 2 of these 3 participants cited high costs associated with mental health services as a major barrier. Furthermore, all 3 participants compared Appa Health favorably to traditional teenage mental health services, indicating that the web-based nature of Appa Health provides a more convenient option for teenagers. Two of these participants expressed the following:

I would say I feel even better about Appa [after completing the program]. Before the program I like, did in person therapy before and honestly, like, it's a big commitment to like, drive to and like, take that time out of your day. So it was really nice for [Appa] to just, you know, be in the comfort of your own home, and do things more remotely.

Cisgender male participant, 18 years old

It's hard to find support for people that are under 16. And I think it's also hard to find support that's not going to cost like $400 for session. And I also, I had found a therapist, but she just was more trained with speaking to adults. So it wasn't really working out for me. And I couldn't make the hour-long sessions with my school schedule. So...the help that's there sometimes doesn't work. And so I just don't think there was enough. But then [with Appa], I thought, well, it's like, only 30 minutes, and I can talk to my mentor whenever and the videos I can watch and I can look back on. And I just realized it was a lot more helpful than what I had before.

Transgender female participant, 15 years old

### Appropriate Candidates for Appa Health

When asked who might be an appropriate candidate for Appa Health, 3 participants indicated that Appa would be a good fit for teenagers who are “willing to share” and are “open-minded” about discussing their experiences and getting to know their mentor.

One participant expressed that Appa Health feels geared more for teenagers who are more self-aware of their emotional state and need extra support in their daily lives. However, the participant indicated that a therapist should be used for more professional help:

I think a teenager who is aware of their emotions. I don't think it's for people who desperately need professional help, but it's more of a thing for like, people who want extra support. Because if you need professional help, you should go to a professional therapist. But if you need like, just a little bit of support in your daily life and talk to somebody that I think this is great for you.

Cisgender male participant, 18 years old

Lastly, one participant with experience with traditional mental health services indicated that Appa Health would be appropriate for teenagers who may not want to have a traditional therapy session with a therapist due to time commitments or challenges like anxiety. This participant said:

I think [Appa would be good for] someone who's looking for someone to talk to you that's not sitting there writing on a notepad taking down everything you say, and someone it's not trying to analyze all of your thoughts. Also [for] someone who's struggling through something, or if they're just kind of getting stressed a lot or getting anxious a lot. I would say someone who's more likely, like doesn't have time for longer sessions.

Transgender female participant, 15 years old

### Appa Health Mentoring Experience and Dynamics

All 5 participants expressed having had a highly positive experience with their mentors. Participants all described their mentors as compassionate individuals who were able to listen to their struggles and current situations and offer them constructive advice, support, and a space for understanding and dialog. All participants expressed looking forward to their mentoring sessions. One participant reported applying lessons she learned from her mentor when she was experiencing a difficult time:

When I started the program, I was in a, like, a really low spot. And I think that my mentor, like, she really kind of helped me through that.

Cisgender female participant, 12 years old

The participant went on to explain that her mentor leveraged her own lived experience to demonstrate a technique to shift her perspective when negative events occur. This simple technique entailed acknowledging the presence of negative events while simultaneously noticing positive events that are also present in her life. The mentor’s explanation parallels Appa’s content and further reinforces the concept.

Another participant described challenges with feeling comfortable openly sharing with others. However, through the mentoring component of Appa Health, this participant recalled being able to share more candidly with her mentor. She said:

I struggled talking about the past [but] like towards the end of the program.... I knew my relationship was close with my mentor. And I trusted her.... I was able to, like open up to her. And she was able to like guide me through it sort of, even though like it was something that had already happened, but like had still been affecting me. She was just powerful.

Cisgender female participant, 17 years old

Finally, a participant expressed valuing the ability to select a mentor that could relate to their racial identity, saying:

When we first started, we had the option to request for a mentor to be a person of color. And I just really liked that option. It just made me a lot more comfortable sharing my experiences.

Cisgender male participant, 18 years old

In total, 3 of 5 participants reported missing the mentoring component of Appa Health, now having completed the curriculum. These participants all expressed feeling “sad” at not being able to connect with their mentors on a weekly basis as they had done while in the program, saying:

I was really excited every week to like, get to talk to her. And she was also just like, really nice.... So it was like really sad actually having to like, have our last call and everything.

Cisgender female participant, 12 years old

I'm actually really sad that it ended, because I'm really glad that I got the opportunity to join. And I would not change that at all. But I am very sad...because sometimes things will happen and I'm like, “Oh, I wish I could text my mentor right now.”

Transgender female participant, 15 years old

### Appa Health Video Experience

All participants expressed positive attitudes toward the Appa Health program short-form CBT skill videos. Participants all reported liking the length of the videos, expressing that it facilitated engagement with the content:

I really liked it because it was really informational and a lot of new things and new concepts but also like compressed into one-minute to two-minute video that was easily digestible. I wouldn't have to like, get bored or anything like that. Like I would be understanding and engag[ing] with the video.

Cisgender male participant, 18 years old

One participant suggested that if longer videos were made for the program, a short video summary should still be included:

I did really like the bite-sized videos because I think like I started getting a lot of schoolwork.... I don't think I could watch an hour-long video. I think like, the short videos were [a] really good size. But if they were looking to do longer videos, maybe still include some like, like a summary video that could go along with it that people could watch.

Transgender female participant, 15 years old

In terms of recommendations for video content and engagement, one participant recommended that mentors use the videos to further engage with participants on weekly content and discussions while in the Appa Health program. This participant stated:

It would be helpful if like the mentors also went over everything with you just to like, clarify everything and like maybe go into more detail about it.

Cisgender female participant, 12 years old

Other participants recommended that the videos expand to further share more “personal experiences” or to illustrate more longer-term approaches to understanding and handling one’s mental health. This participant stated:

I think [the videos] did cover...how to handle your emotions very well. But I feel like, I feel like there wasn't really like approach to like, fixing it, it was more of an approach of like, helping you right then and there to like, calm down, and think about the situation more. The videos didn't really feel like, “Hey, if you do this, you could work on your mental health from here.”...I just wanted something more.

Cisgender male participant, 18 years old

Lastly, one participant also recommended including video content on feeling “overwhelmed” and “overworked” due to school commitments and demands.

### Applications of Appa Health Learned Skills and Techniques Into Everyday Life

When asked if and how the skills learned through the Appa Health program were being applied into their everyday lives, all participants said they benefited from learning through the Appa Health program and that they used the information and instructions gained from the Appa Health videos or their mentorship relationships to address their own mental health. In one example, a participant recalled using the *cognitive behavioral triangle* to address negative thoughts:

I definitely use the...behavioral triangles. So like, if I'm feeling a certain way, I try and trace it back to why. And then if I can't really change my thought on why, like if I can't really change my negative thoughts, and I try and change behavior to hopefully influence my emotions.

Transgender female participant, 15 years old

In another example, a participant discusses using CBT learned through their mentor to address experiences of anxiety at school:

So at my school I’m on a leadership team, so I had to do announcements one day, and that really was triggering my anxiety because I was anxious about it. But [my mentor and I] talked about like CBT and like changing our thought processes. So I just thought about that and what [my mentor] had talked about, and about, like how to change these like negative thoughts that might not even be realistic or like the reality so I thought about that, and that helped me a lot to get over that fear.

Cisgender male participant, 18 years old

Lastly, one participant recalled applying breathing techniques learned through Appa Health when feeling stressed and anxious:

I can get really like stressed out. I'm in track, and yesterday, we had an away meet, which meant that we had to ride the bus because my parents didn't get home in time to like, drive me there.... I get really stressed out on buses, so I was using the breathing techniques [and] that really helped me out yesterday. Also, when I get really anxious at night, too.... I use the breathing techniques, then.

Cisgender female participant, 12 years old

## Discussion

### Principal Results

The purpose of this study was to use adolescent participant data from Appa Health, a 12-week web-based mentoring program combining weekly 30-minute video sessions, text chat, and a structured curriculum of short-form videos teaching CBT skills, to determine acceptability and preliminary efficacy for mental health and well-being of youth.

Teenagers’ primary reasons for joining Appa related to mood, stress, and anxiety. A qualitative examination of participants’ top problem areas indicated that problems most often cited by teenagers were related to performance and interpersonal concerns.

Feasibility and acceptability measures of the present participants suggested that the Appa Health program was feasible with satisfactory rates of engagement with mentors via video chats. Participants also found the program acceptable: MYAS scores were high, and the overall NPS was excellent, with around 85% (n=12) of young people choosing a 9 or 10 out of 10 when asked “how likely is it that you would recommend Appa to a friend?” Qualitative data from interviews enriched the picture of feasibility and acceptability. Participants were enthusiastic about Appa, citing the reduction of barriers to care, highly positive mentor experiences, and appreciation of both the content and presentation of the short-form videos. Youth suggestions for improvement of video content included additional content on feeling overwhelmed by schoolwork and longer-term approaches to improving and sustaining mental health.

Youth scores on measures of symptoms of depression and generalized anxiety demonstrated promising trends over the course of the program with scores on the PHQ-8 and the GAD-7 reducing in severity over the course of the 12-week program.

### Comparisons With Prior Work

This is the first study assessing the combination of short-form digital content and near-peer mentoring from mentors with shared lived experiences of youth depression and anxiety. While this concept is novel, there is precedent for both the core concept of pairing human support with digital tools [21-23] and for mentors supporting the delivery of mental health interventions [11-16]. While this design does not allow us to compare to young people receiving digital tools or human support alone, the preliminary quantitative and qualitative results here indicate that youth with significant symptoms of depression or anxiety felt adequately supported by their mentor and by the CBT content and that the human support was crucial to the feasibility and acceptability of the intervention. This is consistent with prior literature.

### Limitations

While this study has several strengths, including a theory-driven and evidence-based approach drawing on CBT and SCT and in-depth qualitative interviews to provide a fuller picture of feasibility and acceptability, it is important to acknowledge several limitations of this pilot study that require additional research. First, due to low response rates from parents and guardians as the Appa team sought research consent, the overall sample of teenagers who consented to participate in the research was limited. Thus, a self-selection bias toward more engaged teenagers and their parents is likely. The teenagers in this study consented or assented to research and completed measures at all 3 timepoints. These teenagers may be qualitatively different than those who were not able to participate in this study. Second, due to the small sample size, the ability to explore greater nuanced experiences in Appa Health was limited. An expanded sample will allow for the exploration of characteristics of teenagers who are engaged in Appa and for whom the program works, providing more clarity on mechanisms of effectiveness. Third, the lack of follow-up post–end point survey means we are unable to assess sustained improvements. Lastly, there was no objective metric of engagement with the CBT videos. As such, there are many potential areas for future investigation. Running a similar investigation using a larger, more diverse sample of teenagers and incorporating additional measures of feasibility, such as rates of recruitment, adherence, and attrition, would be helpful in expanding upon this pilot’s results. Additionally, examination of reasons for and patterns behind attrition will be necessary to better understand who is best suited for participation. Finally, large-scale randomized controlled trials would be helpful in further understanding the effectiveness of this novel solution (short-form CBT skills videos coupled with paraprofessional mentorship) relative to its components (standalone CBT and standalone mentorship) as well as existing other solutions (therapy and other types of digital tools), in addition to further understanding the underlying mechanisms of action. It also remains to be seen where an intervention like Appa Health fits on the care continuum in the broader digital mental health landscape. Nevertheless, this study provides directional evidence of a potentially clinically meaningful and engaging intervention to meet the mental health needs of adolescents.

### Conclusions

Appa Health aims to be part of the solution to the youth mental health crisis by combining brief video-based CBT delivery methods in a modern, accessible format to adolescents with evidence-based mentoring for teenagers with depression and anxiety, all delivered remotely in a fully digital environment. Appa’s approach to video production uses a format with proven engagement success (TikTok influencers with clinical expertise and a significant social media following), and Appa’s approach to mentoring is theory-driven and evidence-based, leveraging near-peer mentors with shared lived experience and identities who are trained to provide supportive accountability. Moreover, the use of paraprofessionals (trained mentors supervised by clinical psychologists) and auxiliary methods (video) to supplement, support, or supplant the clinical workforce could provide a transferable, generalizable model to increase access to effective services and support overburdened professionals. Finally, Appa’s large, diverse pool of remote mentors permits a wide sample of near-peers with whom to match young people and foster alliance. In sum, Appa Health has the potential to become a blueprint for accessible, mentor-supported, self-help prevention and intervention strategies.

## Abbreviations

CBT: cognitive behavioral therapy
GAD-7: Generalized Anxiety Disorder-7
LGBTQ: lesbian, gay, bisexual, transgender, queer
MYAS: Mentor-Youth Alliance Scale
NPS: net promoter score
PHQ-8: Patient Health Questionnaire-8
SCT: social cognitive theory
TPA: Top Problems Assessment

## Data Availability

Data generated and analyzed in this study are not publicly available due to Appa Health’s internal privacy and security policies, as well as ethical restrictions relating to participant consent. Appa strives to ensure the trust of its customers—especially parents and adolescents—by honoring these policies.
